# Molecular identification and functional characterization of the first Nα-acetyltransferase in plastids by global acetylome profiling

**DOI:** 10.1002/pmic.201500025

**Published:** 2015-06-18

**Authors:** Trinh V Dinh, Willy V Bienvenut, Eric Linster, Anna Feldman-Salit, Vincent A Jung, Thierry Meinnel, Rüdiger Hell, Carmela Giglione, Markus Wirtz

**Affiliations:** 1 Department of Plant Molecular Biology, Centre for Organismal Studies, University of HeidelbergHeidelberg, Germany; 2 Institute of Integrative Biology of the Cell (I2BC), CEA, CNRS, Université Paris-SudGif-sur-Yvette, France; 3 Hartmut Hoffmann-Berling International Graduate School, University of HeidelbergHeidelberg, Germany; 4 Molecular and Cellular Modeling Group, Heidelberg Institute for Theoretical Studies gGbmHHeidelberg, Germany

**Keywords:** *Arabidopsis thaliana*, AtNAA70, Chloroplast, N^α^-acetyltransferase, Plant proteomics

## Abstract

Protein N^α^-terminal acetylation represents one of the most abundant protein modifications of higher eukaryotes. In humans, six N^α^-acetyltransferases (Nats) are responsible for the acetylation of approximately 80% of the cytosolic proteins. N-terminal protein acetylation has not been evidenced in organelles of metazoans, but in higher plants is a widespread modification not only in the cytosol but also in the chloroplast. In this study, we identify and characterize the first organellar-localized Nat in eukaryotes. A primary sequence-based search in *Arabidopsis thaliana* revealed seven putatively plastid-localized Nats of which AT2G39000 (AtNAA70) showed the highest conservation of the acetyl-CoA binding pocket. The chloroplastic localization of AtNAA70 was demonstrated by transient expression of AtNAA70:YFP in Arabidopsis mesophyll protoplasts. Homology modeling uncovered a significant conservation of tertiary structural elements between human HsNAA50 and AtNAA70. The in vivo acetylation activity of AtNAA70 was demonstrated on a number of distinct protein N^α^-termini with a newly established global acetylome profiling test after expression of AtNAA70 in *E. coli*. AtNAA70 predominately acetylated proteins starting with M, A, S and T, providing an explanation for most protein N-termini acetylation events found in chloroplasts. Like HsNAA50, AtNAA70 displays N^ε^-acetyltransferase activity on three internal lysine residues. All MS data have been deposited in the ProteomeXchange with identifier PXD001947 (http://proteomecentral.proteomexchange.org/dataset/PXD001947).

## 1 Introduction

N^α^-terminal acetylation (NTA) is one of the most common protein modifications in eukaryotes, occurring on 80–90% of cytosolic human proteins and 50–70% of cytosolic yeast proteins [1,2]. However, the biological role of this modification has remained enigmatic. In few cases, NTA has been shown to affect protein–protein interaction and subcellular targeting [1,3,4]. NTA has been suggested to stabilize protein by blocking ubiquitination mediated degradation in the N-end rule pathway [5]. On the contrary, some acetylated N-termini can be recognized by Doa10, a ubiquitin ligase that targets proteins for proteasome degradation in yeast [6]. Very recently, also aggregation has been linked to N-terminal acetylation status of proteins [7].

NTA is catalyzed by N^α^-acetyltransferases (Nats) which transfer the acetyl group from acetyl-coenzyme A (acetyl-CoA) to the α-amino group of the N-terminal amino acid of a protein and is considered to be irreversible. The acetylated amino acid may be the initiator methionine (iMet) or the first residue after the removal of iMet by methionine aminopeptidases [8]. This co-translational modification occurs when 10–70 residues have left the exit tunnel [9,10]. Eukaryotic Nats usually consist of a catalytic subunit and sometimes, an auxiliary subunit suggesting to favor interaction with other Nat binding partners and/or to anchor the complex to the ribosome [1,11,12], e.g. NatA consists of the catalytically active NAA10 and the auxiliary subunit NAA15. At present five catalytic subunits of Nats (ScNAA10-ScNAA50) are known from yeast and six from humans (HsNAA10-HsNAA60) of which NAA10 to NAA50 orthologous are conserved with respect to amino acid sequence, cytosolic localization and substrate specificity [1,13,14].

In plants, besides the co-translational acetylation of cytosolic proteins, NTA is reported to be a widespread modification of chloroplastic proteins from both origins, i.e. those deriving from the plastid genome expression and plastoribosomes translation (e.g. RbcL and CP43; see http://www.isv.cnrs-gif.fr/recherche/tm/maturation/images/chloro.html and [15,16]) and the nuclear-encoded proteins which are further imported in the plastid [17–20]. This implies that NTA occurs not only co-translationally for plastid-encoded proteins as in the cytosol but also post-translationally for plastid-imported proteins of photosynthetic organisms [20]. Furthermore, the post-translational acting Nat(s) might also (in part) be responsible for the N-terminal acetylation of plastid-encoded proteins. Interestingly, NTA of the chloroplastic ε subunit of ATP synthase protein was evidenced to regulate its stability during drought stress [21]. Similarly, the stability of the plastid proteome is dependent on the NTA status and other N-terminal modifications in the green algae *Chlamydomonas reinhardtii* [22]. Despite the importance of NTA in the chloroplast for regulation of protein turnover, the NTA machinery of chloroplastic proteins in general is uncharacterized (i.e. co- and/or post-translational NTA). Only cytosolic AtNAA20 and AtNAA30 from *Arabidopsis thaliana* have so far been functionally analyzed in higher plants [23,24]. In this study, a homology based in silico search of the Arabidopsis genome revealed 25 putative Nats, of which seven are predicted to be localized in the chloroplast. Subcellular localization in Arabidopsis protoplasts and the use of a new global acetylome profiling (GAP) approach clearly demonstrate that AtNAA70 displays indeed N^α^-acetyltransferase activity. The substrate specificity of AtNAA70 was even broader than substrate specificities of cytosolic metazoan Nats. Pairwise alignment and modeling of AtNAA70 to cytosolic human NAA50 (HsNAA50) allows identification of the catalytically active site of AtNAA70 and reveals a remarkable conservation of the tertiary structure between post-translationally acting AtNAA70 and co-translationally cytosolic metazoan Nats. Interestingly, AtNAA70 possesses, like HsNAA50 and HsNAA10, auto-Lys^ε^ acetylation (Kat) activity.

## 2 Materials and methods

### 2.1 Three-dimensional structure modeling of N^α^-acetyltransferases from Arabidopsis

Local sequence pairwise alignments of putative Arabidopsis Nat encoded by loci AT2G39000 (Q9ZV08) against human HsNAA50 (2PSW, Q9GZZ1) were performed with BLASTP 2.2.30 [25]. A tertiary structure was defined for the regions at residues 63 –112 and 195 –254 of AT2G39000 by comparative modeling against the resolved quaternary structure of 2PSW with MODELLER9.1 (applied settings: “automodel.very_fast”, [26]). Conservation analysis and visualization was performed with the UCSF Chimera package [27]. Functional protein domains were named according to the Conserved Domains Database (CDD, http://www.ncbi.nlm.nih.gov/Structure/cdd/cdd.shtml).

### 2.2 Construction of plasmids

Total RNA was isolated with the RNeasy™ Plant Mini Kit (Qiagen) from Arabidopsis leaves and reverse transcribed to cDNA with the RevertAid H Minus First Strand cDNA Synthesis Kit (Fermentas). The AtNAA70 sequence was amplified from cDNA with specific primers containing appropriate restriction endonuclease sites (Supporting Information Table 1) using Phusion High fidelity DNA Polymerase (Finnzymes). The cDNAs encoding for full-length AtNAA70 was cloned in pFF19-EYFP, which allowed expression of AtNAA70 fused with EYFP at the C-terminus. The cDNA encoding for AtNAA70 lacking the predicted transit peptide was cloned in the pETM41 vector to allow expression of His_6_-MBP-AtNAA70 in *E. coli*.

### 2.3 Subcellular localization of AtNAA70

Transformation of isolated protoplasts from rosette leaves of six-week-old *Arabidopsis thaliana* ecotype Col-0 was performed according to [28] using 10–20 μg of pFF19-AtNAA70:EYFP DNA. Protoplasts expressing AtNAA70:EYFP for two days were placed on a slice in a drop of 20 μl WI solution and imaged using Zeiss confocal microscope LSM510META equipped with lasers for 405-, 488- and 543-nm excitation. Images were documented with 63x lens in multi-tracking mode. YFP fluorescence was excited at 514 nm and emission was recorded with a 560–615 nm band-pass filter. Chlorophyll auto-fluorescence was excited at 488 nm and emission was collected within 647–745 nm. Images were analyzed with the LSM 510 software suite (Zeiss).

### 2.4 Expression and purification of AtNAA70 in *E. coli*

*E. coli* Rosetta (DE3, Novagen) was transformed by electroporation with the pETM41-AtNAA70 plasmid encoding for a His_6_-MBP-AtNAA70 fusion protein, and grown overnight on LB plate containing kanamycin. The His_6_-MBP-AtNAA70 protein was expressed and purified by immobilized metal affinity chromatography as described in [29].

### 2.5 In vitro Lys-auto-acetylation assays

In vitro auto-acetylation assay was performed according to [30] with two independent methods. For determination of lysine acetylation with a N^ε^-Lyse specific antiserum, 1 μg of purified His_6_-MBP-AtNAA70 was incubated in a total volume of 80 μL reaction buffer (50 mM Tris-HCl (pH 8.5), 10% glycerol, 1 mM EDTA, 125 μM Acetyl-CoA). The degree of lysine acetylation was quantified (auto-Kat activity) by immunological detection using a specific anti-acLys antiserum (New England Biolabs, 1:10.000), the anti-Rabbit IgG-HRP antiserum (Promega; 1:20.000) and the SuperSignal™ West Dura Extended Duration Substrate (Thermo Scientific). Additionally, the auto-Kat activity of AtNAA70 was quantified by determination of incorporated [^3^H] label with a Tri-Carb 2810TR scintillation counter (PerkinElmer) after incubation of 20 μg purified His-MBP-AtNAA70 at 37°C in acetylation buffer (50 mM Tris-HCl (pH 8.5), 10% glycerol, 1 mM EDTA) containing 60 μM [^3^H]-acetyl-CoA (7.4 GBq/mmol, Hartmann Analytics). Free [^3^H]-acetyl-CoA was removed after incubation with a PD-SpinTrap (GE Healthcare) according to the manufacturer's protocol.

### 2.6 Sample preparation and N-terminus peptide enrichment

Bacterial cells over-expressing AtNAA70 were centrifuged at 5000 rpm 4°C and pellets frozen at –80°C. The cell pellets were resuspended in 50 mM Tris pH 8 plus 250 mM NaCl, subjected to sonication and cell debris were removed by centrifugation at 15 000 rpm for 30 min at 4°C. 1 mg of supernatant proteins was denaturated and reduced before cysteine alkylation using iodoacetamide [19]. Then, to perform stable isotope labeling protein N-terminal acetylation quantitation (SILProNAQ), the sample was chemically treated to acetylate the free amino groups with d3-acetyl groups [31]. Rapidly, 25 μmol of N-acetoxy-[^2^H_3_]-succinimide in DMSO per mg of protein was added to the alkylated proteins resuspended in 50 mM phosphate buffer at pH 7.5. After 90 min at 30°C, potential O-acetylation of Ser, Thr and Tyr side chains were reversed by adding 10 μl of 50% of hydroxylamine (v/v) and incubated for 20 min at room temperature. After a purification step using cold acetone precipitation, the protein pellet was resuspended in 50 mM ammonium bicarbonate and digested by the addition of 1/100 (w/w) of TPCK treated porcine trypsin (Sigma-Aldrich) for 1.5 h at 37°C twice. Peptides were desalted by Sep-Pak™ solid phase extraction and the retained material was eluted with 80% ACN, 0.1% TFA followed by evaporation to dryness. The collected material was resuspended in strong cation exchange (SCX) LC buffer A (5 mM KH_2_PO_4_, 30% ACN and 0.05% formic acid) and injected into Summit LC system (Dionex, Sunnyvale, CA) equipped with Polysulfoethyl A 200 × 2.1 mm 5 μm 200 Å column (PolyLC, Colombia, MD). Peptides were eluted with a gradient of increasing KCl concentrations (SCX-LC Buffer B: 350 mM KCl in SCX-LC Buffer A; 0–5 min, 0% B; 15–40 min, 5–26% B; 40–45 min, 26–35% B). Fractions were collected every 2 min for 40 min and solvent was evaporated to dryness before storage at –20°C.

### 2.7 LC-MS/MS analysis

Analysis of the SCX-LC fractions was performed as previously described [32]. Rapidly, selected samples are loaded on the pre-column (NS-MP-10, Nano-separation,) of the Easy nLC II system (Thermo Scientific) followed by the separation on a C18 reverse phase column (NikkyoTechnos capillary column,) at a flow of 300 nL/min on 40 min gradient. The nano-LC was coupled to an Orbitrap™ Velos (Thermo Scientific). The survey scan was acquired by Fourier-Transform MS scanning 400–2000 Da at 30 000 resolution using internal calibration (lock mass) using the Top-20 acquisition method with 20 s exclusion time. Raw data files were extracted and exported with Proteome Discoverer (Thermo Scientific, Ver. 1.4) for ion signal higher than 1 counts and S/N higher than 1.5.

### 2.8 Data processing for protein identification and quantification

MASCOT 2.4 software was used for protein identification and co-/post-translational modification characterization using the *E. coli* K12 strain reference proteome subset extracted from UniProtKB (version 112) which also included the sequence of the recombinant proteins AtNAA10, AtNAA15 and AtNAA70. Trypsin/P rules were used with parent and fragment mass tolerance defined as 10 ppm and 0.7, respectively. Carbamidomethylcysteine and d3-acetyl on Lys were considered as fix modifications whereas Met-oxidation, protein NTA and d3-NTA were considered as variable modifications. All data were filtered at 1% protein false discovery rate and only peptides with score higher than 25 are retained for the final data treatment. To extract specifically N-terminal peptides, MASCOT searches were exported in xml format and submitted to an in-house script written in Python. The parsing function searched for modifications and collected peptides with defined modifications such as d0/d3-NTA.

SILProNAQ quantification required the aid of MASCOT Distiller (ver. 2.5.1, Matrix Science) to combine the processing of the raw data and MASCOT identification results. MASCOT distiller extraction parameters were optimized for the OrbiTrap “Top-20” acquisition files with a minimum S/N of 1, precursor charge from 1–5, Corr. Thr. > 0.7 and no grouping assignments. MASCOT distiller submissions for protein identification were performed using the MASCOT 2.4 identification tool against the reference proteome for *E. coli* K12 strain extracted from UniProtKB (version 112) using Trypsin/P cleavage rules. The parent and fragment mass tolerance were defined to 10 ppm and 0.7 Da, respectively, with carbamidomethylcysteine and d3-acetyl on Lys as fix modifications. Few variable modifications were used including oxidized Met (Met-Ox) and d0/d3-NTA quantitation method. Based on characterized proteins, MASCOT distiller determined NTA quantitation from the raw data. Since this tool was designed to deliver quantification values at the protein level (including all characterized peptides and not the protein N-terminus only), we developed an in-house Python script able to parse MASCOT distiller xml export files and to recalculate the NTA yield based only on the data related to the N-terminus peptides sharing a common starting position.

This tool extracts a few parameters including H/L ratio, signal quality coefficient (MASCOT distiller defined parameters: fraction, *E* value, correlation) and peptide related data (peptide start position, MASCOT score, peptide mass error). For each distinct N-terminus position, EnCOUTer aggregates the data passing the filtering criteria defined by the user and especially the signal quality coefficient (Corr > 0.5, *E* value < 0.1, score > 30, std. err. < 0.1, fraction > 0.5). Then, the geometric mean and geometric deviation was re-calculated including all data associated with the same N-terminus position that pass the filtering criteria defined by the user. H/D value is finally converted to NTA yield. Filtering parameters defined and provided by MASCOT distiller were also used in the extraction script, i.e. *E* val. < 0.1, std. err. < 0.1, MASCOT score > 30, corr. > 0.5, fraction > 0.5, Sc. *P* > 0.3. NTA yield was determined from the d0/d3 ratio and expressed in % of NTA for the different N-termini.

## 3 Results

### 3.1 Identification of potential chloroplastic N^α^-acetyltransferases in *Arabidopsis thaliana*

Several studies demonstrated the presence of acetylated N-termini from plastid localized proteins [18–20]. However, the few characterized plant Nat proteins are all localized in the cytosol [19,23], and thus could not be responsible for the N-terminal acetylation of these proteins.

In a search of plastidic Nats, sequences of known Nats from human, yeast and Arabidopsis were used as templates in a blast search on TAIR to find unknown Nat candidates [13,14,23,33]. This sequence homology based search revealed 25 putative Nats in the Arabidopsis genome (Supporting Information Fig. 1). According to the SubCellular Proteomic Database (http://suba.plantenergy.uwa.edu.au/) seven of the 25 candidates are predicted to localize in chloroplast (listed in Supporting Information Table 2). The protein encoded by AT2G39000 displayed the highest conservation of the acetyl-CoA binding motif RxxGxG/A, which is conserved in all major Nats of eukaryotes [34]. Transcription of the AT2G39000 gene has been verified by qRT-PCR in roots, stem, flower, rosette and cauline leaves as according to [35] and was found to be approximately five-fold higher in rosette and cauline leaves when compared to non-green tissues (flower and root). However, AT2G39000 is ubiquitously transcribed during development of *Arabidopsis thaliana* according to public available transcriptome profiling repository databases (Supporting Information Fig. 2).

### 3.2 Tertiary structure modeling of potential chloroplastic N^α^-acetyltransferases

**Figure 1:**
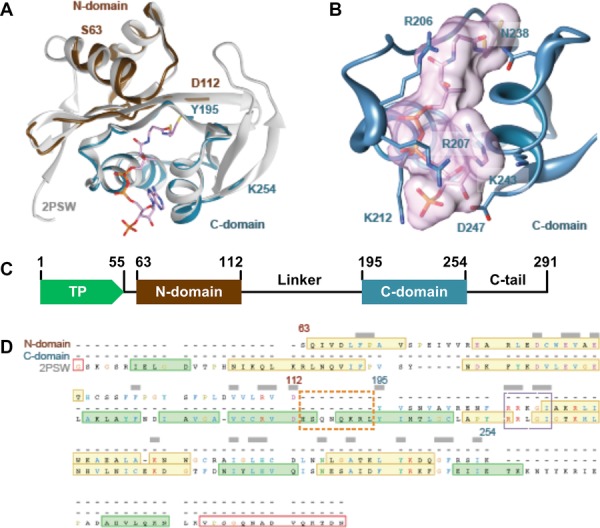
Structure modeling of chloroplastic AT2G39000 from *Arabidopsis thaliana*. (A) Superposition of modeled N-domain (brown) and C-domain (blue) from AT2G39000 with the template 2PSW (light gray) is shown. The enzyme structures are in cartoon representation, whereas CoA molecule is displayed in sticks representation. The modeled domains are labeled by the first and the last modeled residues. (B) Enlargement of modeled AT2G39000 C-domain harboring a CoA molecule (with its molecular surface). The CoA molecule and selected residues that probably interact with CoA are shown in stick presentation. (C) Schematic representation of AT2G39000 domain organization. TP, transit peptide. (D) Structural alignment of N- and C-domains with 2PSW. The orange box highlights the linker of AT2G39000 that is missing in 2PSW. The violet box designates the acetyl-CoA binding motif (RxxGxG/A). Conserved residues are marked by horizontal bars above the sequences. Green areas represent α-helices, yellow areas indicate β-strands.

We decided to use the structural information of HsNAA50 (2PSW) for a modeling approach based on pairwise alignment to gain more information about AT2G39000. After removal of the putative transit peptide domain (residues 1–55) a partial 3D model of AT2G39000 was obtained by modeling residues 63–112 (N-domain, ∼40% similarity to 2PSW) and 195 – 254 (C-domain, ∼45% similarity to 2PSW) to 2PSW, followed by superposition of both domains on 2PSW (Fig.1A). The C-domain included the conserved NAT_SF (N-acetyltransferase family) domain that is known to bind the acetyl-CoA molecule by the RxxGxG/A motif (Fig.1B). Two residues of this motif (R206 and R207) together with charged/polar residues such as K212, N238, K243 and D247 were recognized to contribute to the modeled acetyl-CoA binding pocket of AT2G39000 (Fig.1B). The superimposed N-domain and C-domain of AT2G39000 resemble remarkably the tertiary structure of 2PSW (61% coverage) at the corresponding areas (Fig.1A and D). Thus, we named AT2G39000 according to the revised Nat nomenclature system [13] AtNAA70. The N- and C-domain of AtNAA70 were separated by a linker region that is absent in HsNAA50 (Fig.1C and D).

### 3.3 AtNAA70 is localized in chloroplasts

**Figure 2:**
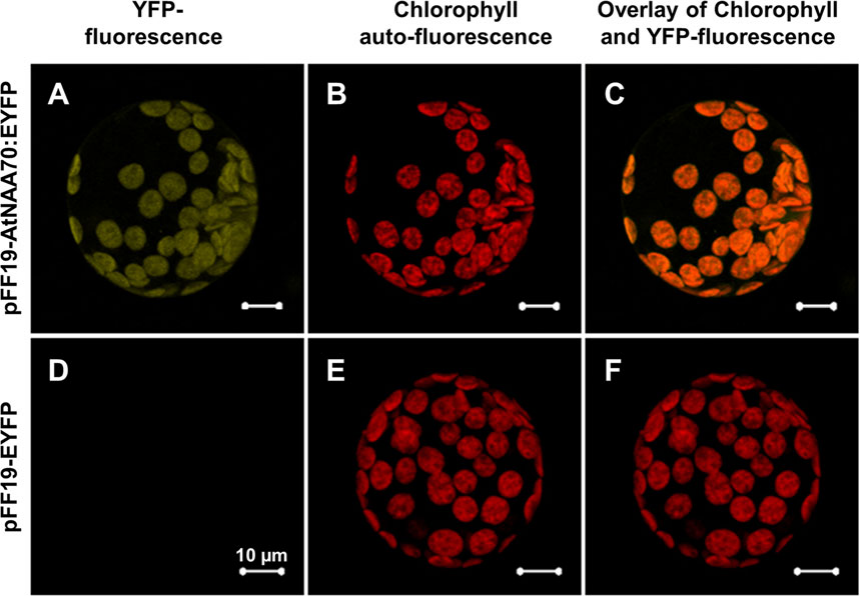
AtNAA70 is localized in chloroplasts. Full length AtNAA70 (AT2G39000) in fusion with EFYP was ectopically expressed under control of the 35S-promotor in mesophyll protoplasts extracted from six-week-old Arabidopsis leaves. The YFP signal (green) of AtNAA70-EYP (A) perfectly overlap (C) with chlorophyll auto-fluorescence (red signal, B) determined in the same protoplasts. Protoplasts transiently transformed with the empty pFF19-EYFP vector (D–F) display chlorophyll auto-fluorescence (E) but no specific EFYP signal (D) using the same settings.

AtNAA70 is predicted by TargetP 1.1 server (http://www.cbs.dtu.dk/services/TargetP/) to target exclusively to the chloroplast (TargetP scores: 0.928, Supporting Information Table 2). In order to confirm its subcellular localization, AtNAA70 fused to EYFP at the C-terminus was transiently expressed in Arabidopsis mesophyll protoplasts. The AtNAA70-EYFP signal perfectly overlaid with chloroplast auto-fluorescence, demonstrating its chloroplastic localization (Fig.2). No YFP signal was detected in control protoplasts expressing the empty pFF19EYFP vector (Fig.2).

### 3.4 AtNAA70 displays N^α^-acetyltransferase activity toward *E. coli* proteins

AtNAA70 lacking the chloroplastic transit peptide (Supporting Information Table 2) in fusion with the 6x histidine-tag and the maltose binding protein (His_6_-MBP) was expressed in *E. coli* cells, where acetylation is a very rare event [1,11,40]. Thus, the comparison of the N-terminal status of the wild type *E. coli* proteome with the proteome of cells expressing His_6_-MBP-AtNAA70 or other plant NAAs should reveal N-termini of proteins acetylated by the recombinant plant proteins. As controls, the already characterized enzymatically active subunit, AtNAA10, and its auxiliary subunit, AtNAA15, of the plant NatA complex were also expressed in *E. coli* as His_6_-MBP-tagged fusion proteins [Linster et al., in revision]. After SCX-LC enrichment, N-termini enriched fractions were analyzed by LC-MS/MS to determine the NTA frequency of the bacterial proteome induced by the expression of the plant Nat subunit. Using this large-scale proteomics approach more than 2000 peptides were identified, which correspond to more than 400 non-redundant protein N-termini in each sample (PRoteomics IDEntifications database, http://www.proteomexchange.org/databases/pride: PXD001947).

**Figure 3:**
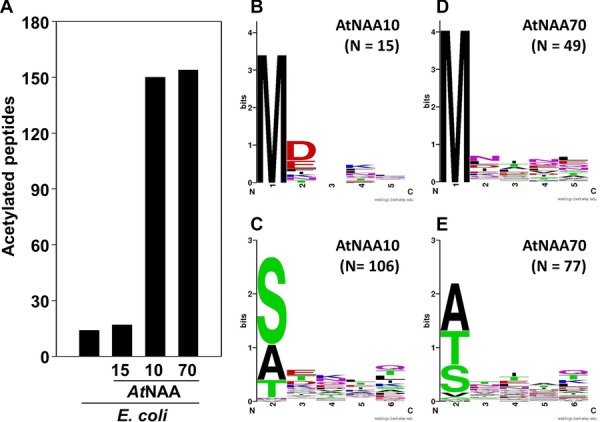
A newly established MS/MS-based in vivo Nat activity test demonstrates enzymatic acetylation activity of AtNAA70. (A) Number of identified acetylated N-terminal peptides after digestion of *E. coli* soluble protein lysates (black bar) with trypsin and enrichment of N-terminal peptides by SCX. Wild type *E. coli* and *E. coli* cells expressing either the auxiliary subunit (AtNAA15, negative control) or the catalytically active subunit of NatA (AtNAA10, positive control) were analyzed to verify the suitability of the new enzymatic Nat test. Expression of catalytic subunit of the NatA and the candidate Nat, AtNAA70, resulted in significant increase of acetylated peptides. (B–E) Web logos of N-termini from *E. coli* proteins that were found to be specifically acetylated after expression of AtNAA10 (B, C) or AtNAA70 (D, E). These proteins start with the iMet (B, D) or were subject of iMet removal (C, E). (*N* = number of acetylated peptides).

As expected only 17 acetylated peptides were identified in the proteome of cells expressing the His_6_-MBP-AtNAA15 (negative control) of which 14 were acetylated also in wild type *E. coli* cells (Fig.3A). In contrast, the proteome of cells expressing His_6_-MBP-AtNAA10 comprised 150 acetylated N-termini of which 121 peptides were specifically found in cells expressing AtNAA10 (Supporting Information Table 3, Fig.3A). The almost 10-fold increase in acetylation of protein N-termini demonstrated the suitability of the here proposed GAP approach for identification of substrate peptides. Only 15 of the 121 uniquely found acetylated peptides in the presence of AtNAA10 starts with an iMet that is predominantly followed by a negatively charged amino residue (Fig.3B). The bulk of acetylated peptides correspond to peptides derived from proteins that were subjected to iMet removal (106, Fig.3C), which matches the in vivo substrate specificity of other eukaryotic NAA10 [36,37]. In an alternative in vitro methodology, a proteome-derived peptide library was used to characterize substrate specificity of purified HsNAA10 (NatA catalytic subunit). This approach evidenced a strong activity of the HsNAA10 against Glu-starting N-termini [37]. This NTA of Glu-starting N-termini by free HsNAA10 could not be observed for free AtNAA10 in the GAP approach. However, we could confirm the promoting function of Glu and Asp residues at position 2 for acetylation of N-termini by free AtNAA10 that was also demonstrated for HsNAA10 with the proteome-derived peptide library approach.

The expression of His_6_-MBP-AtNAA70 also caused a significant increase in acetylation of N-termini of soluble *E. coli* proteins (Supporting Information Table 3, Fig.3A). In the case of His_6_-MBP-AtNAA70 126 specifically acetylated peptides were identified of which 49 start with iMet (Fig.3D). The residual acetylated peptides (77) derived from N-termini that were subjected to iMet removal. The web logo for these peptides demonstrate a preference of NAA70 to acetylate N-termini starting with A > T > S (Fig.3E). The following residue had only minor impact on the specificity of AtNAA70 using the here applied conditions. In order verify the identity of AtNAA70 as a true catalytically active Nat, we purified the protein and confirmed its acetylase activity on protein N-termini according to the here determined substrate specificity (Supporting Information Fig. 3) with an accepted in vitro Nat activity test [36].

**Figure 4:**
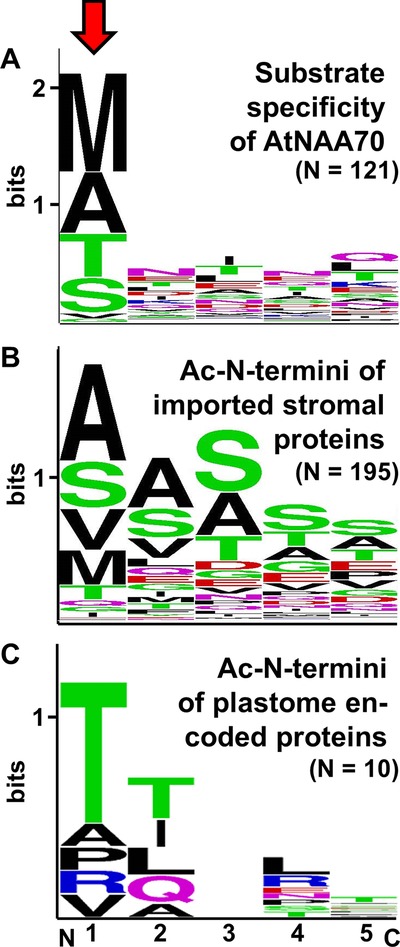
Comparison of AtNAA70 substrate specificity with N-termini of known acetylated plastid localized proteins. Web logo of substrate specificity for His_6_-MBP-NAA70 (*N* = 121, A) is compared with experimentally identified acetylated N-termini of chloroplastic stromal proteins (B, C). The proteins (*N* = 195; [19]) are either imported from the cytosol (C) or translated (*N* = 10) in the chloroplast (D). The red arrow indicates similarity between substrates acetylated by AtNAA70 in *E. coli* and acetylated chloroplastic proteins in Arabidopsis.

Since AtNAA70 should modify the mature N-termini of the imported and plastome-encoded proteins, it was interesting to compare the substrate specificity of AtNAA70 characterized in this study (Fig.4A) with acetylated protein N-termini experimentally found in Arabidopsis chloroplasts (Fig.4B and C). Remarkably, AtNAA70 accepts *E. coli* proteins as substrates that share the N-terminal amino acid with acetylated chloroplastic proteins (Fig.4, arrow). This indicates that AtNAA70 activity contributes to the multitude of N-terminal acetylation events demonstrated in Arabidopsis chloroplasts.

### 3.5 AtNAA70 auto-acetylates residues K217, K254 and K265

**Figure 5:**
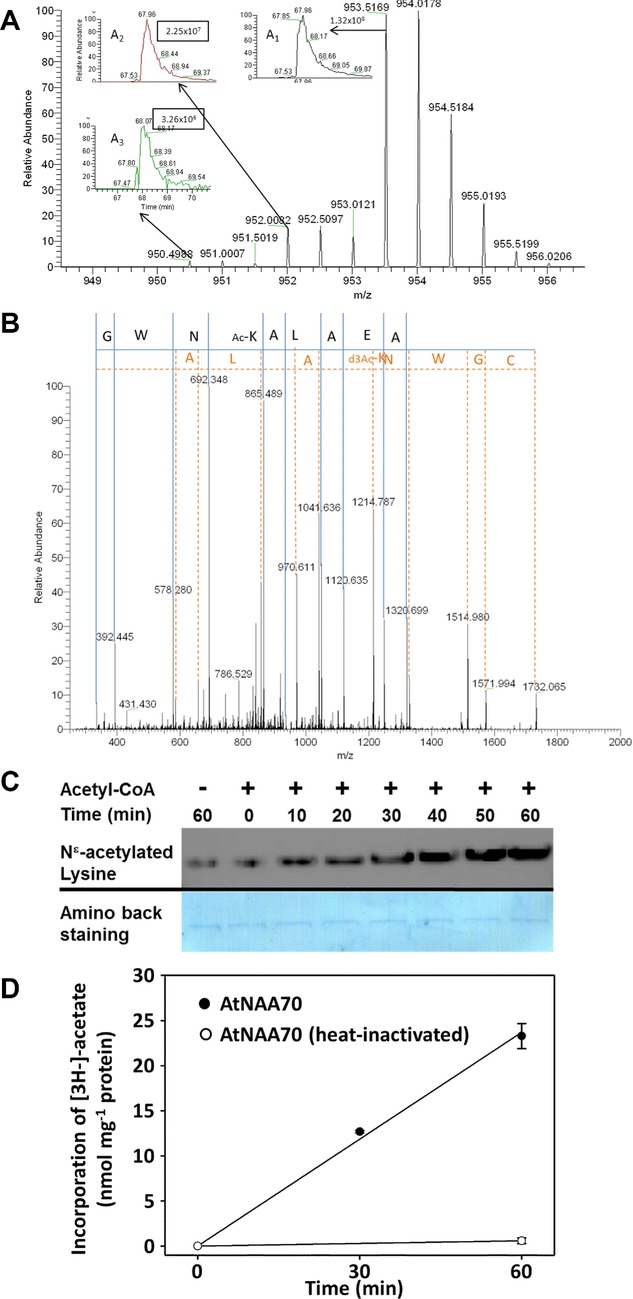
AtNAA70 possesses N^ε^-acetylation activity on internal Lys residues. (A) MS spectrum of the AtNAA70 internal LIWKAEALAKNWGCR peptide with three distinct isotope distributions corresponding to the peptide with A_1_) two heavy acetylations (d3-Acetyl, chemical modification), A_2_) a mix of heavy and light acetylations (d3-Acetyl/Ac), A_3_) two light acetylations (endogenous). The MS signal extractions displayed in A_1_ to A_3_ are used to determine the relative abundance of each form (unmodified peptide: 84%, partial Lys acetylation (on Lys 217 or 223): 14%, Acetylation of both Lys 217 and 233: 2%. (B) Annotated synthetic MSMS of the peptide (combination of spectra with precursor mass of 953.45 ± 0.5 Da). (C) Immunological detection of ac-Lys on AtNAA70 in absence (–) or presence (+) of acetyl-CoA for indicated time points using an N^ε^-acLys specific antiserum. Staining of AtNAA70 with amido black serves as loading control. (D) Determination of AtNAA70 auto-Kat activity by incorporation of [^3^H]-acetyl moieties into purified AtNAA70 (black circles) over time. Incubation of [^3^H]-acetyl-CoA with heat inactivated AtNAA70 (white circles) for 60 min served as a negative control. (*N* = 3)

Since HsNAA50 can acetylate the ε-amino group of Lys residues [30], we screened for additional N^ε^-Lys-acetylated proteins after expression of His_6_-MBP-AtNAA70 in *E. coli*. No significant increase in N^ε^-Lys acetylation was found on *E. coli* proteins. However, three internal lysine residues (K217, K254 and K265) of His_6_-MBP-AtNAA70 were evidenced to be acetylated (Fig.5A and B). In order to test the possibility of N^ε^-Lys auto-acetylation, purified His_6_-MBP-AtNAA70 was incubated with or without acetyl-CoA for different periods of time. Indeed, samples incubated with acetyl-CoA displayed an increase of N^ε^-acetylated Lys residues as determined with a N^ε^-Lys specific antiserum in a time dependent manner, whereas no significant difference between the control sample at time 0 and the sample treated for 60 min without acetyl-CoA was observed (Fig.5C). Signals detected at time point 0 confirm that Lys residues of His_6_-MBP-AtNAA70 are partly acetylated in *E. coli* (Fig.5A). To independently validate the N^ε^-Lys auto-acetylation (auto-Kat) activity of AtNAA70, purified AtNAA70 was incorporated with ^3^H-acetyl-coenzyme A for 60 min. The time dependent incorporation of ^3^H-label into the purified proteins confirmed the determined auto-Kat activity by immunological detection of N^ε^-Lys residues (Fig.5D).

## 4 Discussion

### 4.1 Establishment of an in vivo Nat activity test by global acetylome profiling (GAP test)

Hitherto, substrate specificities of cytosolic Nats have been characterized by large-scale proteomics of respective loss-of-function mutants or by in vitro enzymatic tests using recombinant purified enzymes. Both approaches have specific advantages and drawbacks. Analysis of loss-of-function mutants is the gold-standard to assess endogenous substrates of Nats in vivo [14]. However this approach is not applicable, when the respective Nat is essential in higher eukaryotes or genomic analyses indicates high degree of functional redundancy of gene products [38,39]. The determination of Nat substrate specificity in vitro is hampered by several obstacles: (i) the instability of the purified eukaryotic Nats ([30,36], own observation), (ii) the necessity to provide large numbers of synthetic peptides as substrates [37] and (iii) the lack of high-throughput test systems.

Thus, we designed an in vivo Nat enzymatic activity test that is based on GAP test after recombinant expression of eukaryotic Nat subunits in a heterologous prokaryotic system. In a very similar approach, which was based on expression of HsNAA60 in yeast, the contribution of NatF to the evolutionary shift in N-terminal acetylation from yeast to humans has been analyzed [14]. Furthermore, the HsNAA40 catalytic subunit was biochemically characterized in the yeast *naa40* genetic background by a heterologous expression approach resulting in wild type like complementation [33]. Both approaches were successfully exploited to determine the substrate specificity of eukaryotic Nats, since the orthologous endogenous eukaryotic Nats were absent in the transfected yeast genotype. Like in these approaches the GAP test eliminates the necessity to purify the enzymatically active form of the acetyltransferase and provides a broad range of potential substrates for acetylation. The identification of false positive in vivo N-terminally acetylated substrates is minimized in the GAP test, since the prokaryotic machinery for NTA is limited to a low percentage of substrates of which only a handful were characterized with high NTA yield [1,11,40]. The validation of the GAP test by expressing the AtNAA10 and retrieving the same in vivo substrate specificity known for eukaryotic NAA10 demonstrates its suitability [1,37]. The use of the GAP test can be envisaged for numerous applications. In addition to substrate specificity determinations, kinetic information for the activity of candidate Nats can be obtained by precise pulse-chase experiments. The GAP test has the potential to test for acetyltransferase activity and substrate specificity of candidate proteins in low-throughput screens as a result of its simple design. It allows furthermore screening for potential auto-modifications of the candidate Nat as it has been demonstrated here for AtNAA70 by acetylation of three internal Lys residues. The acetylation of internal Lys has been independently confirmed by immunological detection of the N^ε^-Lys acetylation activity (Kat) of AtNAA70. Kat activity has been also reported for HsNAA10 and HsNAA50 [30,41] and regulates substrate specificity of HsNAA50 [30]. Finally, the GAP test can be applied in future studies to characterize the impact of potential modifiers of Nat substrate specificity, by simple co-expression of the eukaryotic Nat (e.g. AtNAA70) and the potential eukaryotic Nat modifier in *E. coli*. Prime candidates for these modifiers are auxiliary subunits of Nats [42] and ribosome-associated protein biogenesis factors, which are absent or significantly different in prokaryotes when compared to eukaryotes [2].

### 4.2 Structural conservation and substrate specificity of AtNAA70

The in silico analysis of the Arabidopsis genome revealed seven potential candidates for chloroplastic localized Nats. We experimentally confirmed the chloroplastic subcellular localization of one candidate, AtNAA70, unambiguously demonstrated its enzymatic Nat activity and revealed a remarkable structural conservation with a co-translationally acting cytosolic HsNAA50 [2,30]. The tertiary structure of HsNAA50 was re-assembled by two distinct domains (N- and C-domain) of AtNAA70, which are separated by a linker. The assembly of functional structures by modular acting domains is an emerging concept in protein chemistry and contributes significantly to evolutionary driven construction of new protein functions [43]. The C-domain of AtNAA70 harbors the typical GNAT5 acetyl-CoA binding pocket, whose core is formed by a highly conserved RxxGxG/A domain [34].

AtNAA70 has a broad substrate specificity that partly overlaps with all types of known cytosolic Nat complexes (NatA to NatF, [1,2]). The cytosolic Nat complexes are classified by their preference to accept substrates either starting with iMet or with the penultimate residue exposed by iMet removal (non-Met peptides) [1]. HsNAA10 bound in the NatA complex accepts almost solely non-Met peptides. Recently, the interaction of HsNAA15 with HsNAA10 in NatA has been demonstrated to allosterically reconfigure the HsNAA10 active site for sequence-specific N-terminal acetylation [42], which provides a molecular explanation for the fact that free HsNAA10 [37] and free AtNAA10 (this study) are able to acetylate substrates starting with iMet followed by acidic residues (Glu or Asp). Nevertheless, even free NAA10 subunits of both species still prefer to acetylate non-Met peptides ([37], Fig.3B and C). The usage of proteome-derived peptide libraries demonstrated that free HsNAA10 prefers to acetylate glutamic acid at position 1 (82% of all acetylated peptides [37]). Such a substrate preference was not observed for AtNAA10 by using the GAP test, most probably because endogenous proteins starting with Glu are rare in *E. coli*, since Glu at position two inhibits iMet removal in eukaryotic and prokaryotic systems [44].

In contrast, AtNAA70 is able to acetylate iMet and non-Met starting substrates with similar preference in the GAP test (Fig.3D and E). Interestingly, the sole Nat ortholog in the archaea *Sulfolobus solfatarius* (SsNat) is also able to accept both types of substrates, because its active site represents a hybrid of the NatA and the NatE active sites [45]. Since SsNat is believed to represent an ancestral Nat from which the eukaryotic Nat machinery evolved, substrate specification of cytosolic Nat might be a secondary adapted feature during evolution of eukaryotes [45].

As a result of its broad substrate specificity AtNAA70 might be able to acetylate many of N-termini which were experimentally characterized to be acetylated in the chloroplast stroma (Fig.4B and C). However, a minor number of Ile, Glu and Gln starting N-termini might not be acetylated by AtNAA70 according to the here determined substrate specificity (Fig.4A), suggesting the existence of other chloroplastic Nats. Prime candidates for Nats targeting N-termini of proteins starting with Ile, Glu and Gln are the six putatively chloroplast localized Nat-like proteins identified in our homology based bioinformatics screen.

## Abbreviations

Acetyl-CoA: acetyl coenzyme A
AtNAA70: AT2G39000
EYFP: enhanced yellow fluorescent protein
GAP: global acetylome profiling
His_6_-MBP: 6xhistidine-tag-maltose binding protein
iMet: Initial methionine
Kat: N^ε^-acetyltransferase
Nat: N^α^-acetyltransferase
NTA: N-terminal acetylation
SCX-LC: strong cation exchange-LC
SILProNAQ: stable isotope labeling protein N-terminal acetylation quantitation

## Supporting Information

As a service to our authors and readers, this journal provides supporting information supplied by the authors. Such materials are peer reviewed and may be re-organized for online delivery, but are not copy-edited or typeset. Technical support issues arising from supporting information (other than missing files) should be addressed to the authors.
