# Supplementary material for: Molecular identification and functional characterization of the first Nα-acetyltransferase in plastids by global acetylome profiling
Source: Proteomics. 2015 Jun 18;15(14):2426–35. doi: 10.1002/pmic.201500025 (PMC4692087; doi:10.1002/pmic.201500025)
Supplement: Supplementary file 1 — Figure 1 [file pmic0015-2426-sd1.pdf]

PROTEOMICS

Supporting Information for Proteomics

DOI 10.1002/pmic.201500025

Trinh V. Dinh, Willy V. Bienvenut, Eric Linster, Anna Feldman-Salit, Vincent A. Jung, Thierry Meinnel, Rüdiger Hell, Carmela Giglione and Markus Wirtz

Molecular identification and functional characterization of the first Nα-acetyltransferase in plastids by global acetylome profiling

© 2015 The Authors. PROTEOMICS Published by Wiley-VCH Verlag GmbH & Co. KGaA, Weinheim

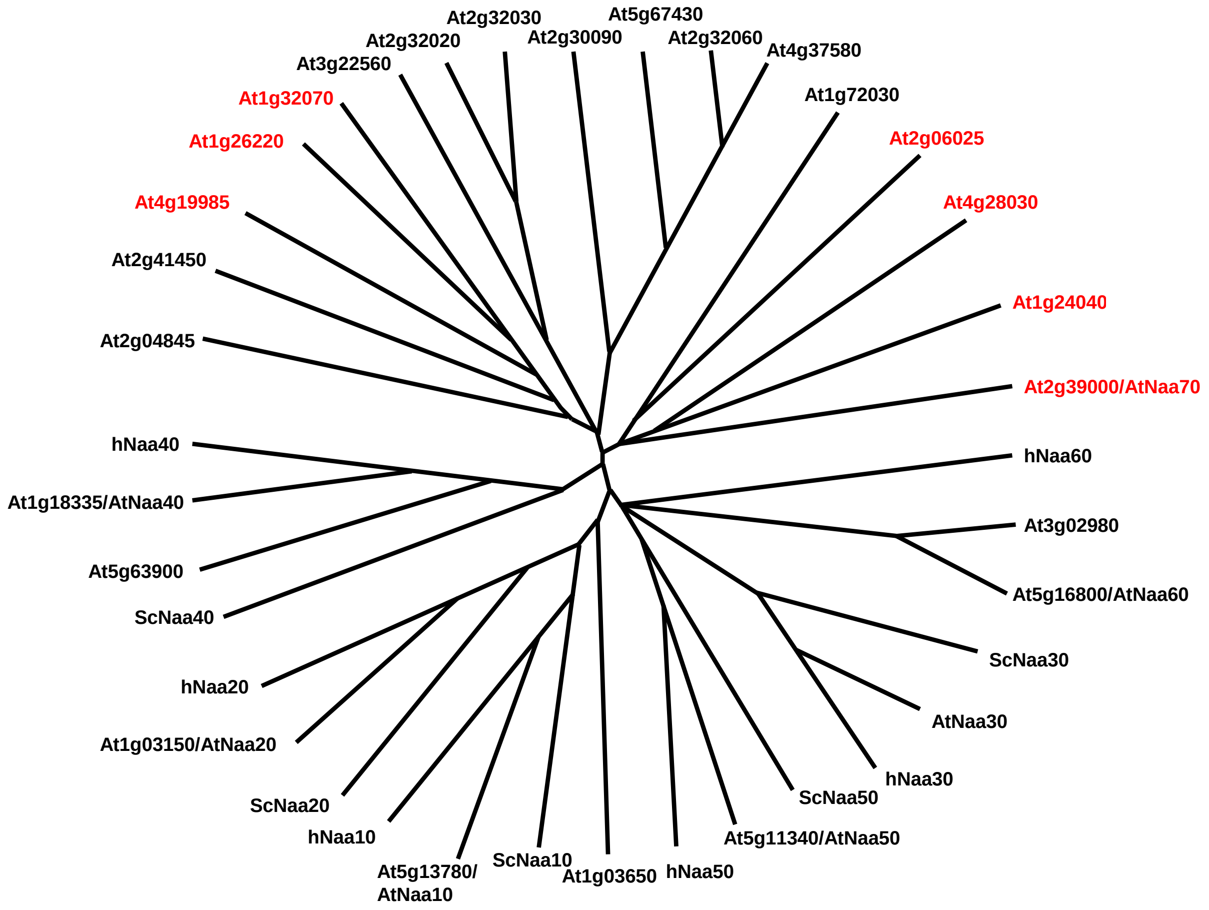

Supplemental Figure 1. Phylogenetic tree of 25 putative Nats present in Arabidopsis
Protein sequences of 12 *bona fide* Nats from eukaryotes have been used to identify potential Nat candidates (25) encoded by the Arabidopsis genome. The phylogenetic tree of known metazoan putative Arabidopsis Nats was created with Clustal Omega [1] and visualized by using the Treeview open software source [2]. Proteins labelled in red are predicted to localize in plastids. Please note that the plant cytosolic Nats cluster with their corresponding Nat from metazoan and that these Nats form a separate branch of the tree (lower part).

References

[1] McWilliam, H., Li, W., Uludag, M., Squizzato, S., *et al.*, Analysis Tool Web Services from the EMBL-EBI. *Nucleic acids research* 2013, *41*, W597-600.

[2] Page, R. D., TreeView: an application to display phylogenetic trees on personal computers. *Computer applications in the biosciences: CABIOS* 1996, *12*, 357-358.
