# Supplementary material for: Molecular identification and functional characterization of the first Nα-acetyltransferase in plastids by global acetylome profiling
Source: Proteomics. 2015 Jun 18;15(14):2426–35. doi: 10.1002/pmic.201500025 (PMC4692087; doi:10.1002/pmic.201500025)
Supplement: Supplementary file 2 — Figure 2 [file pmic0015-2426-sd2.docx]

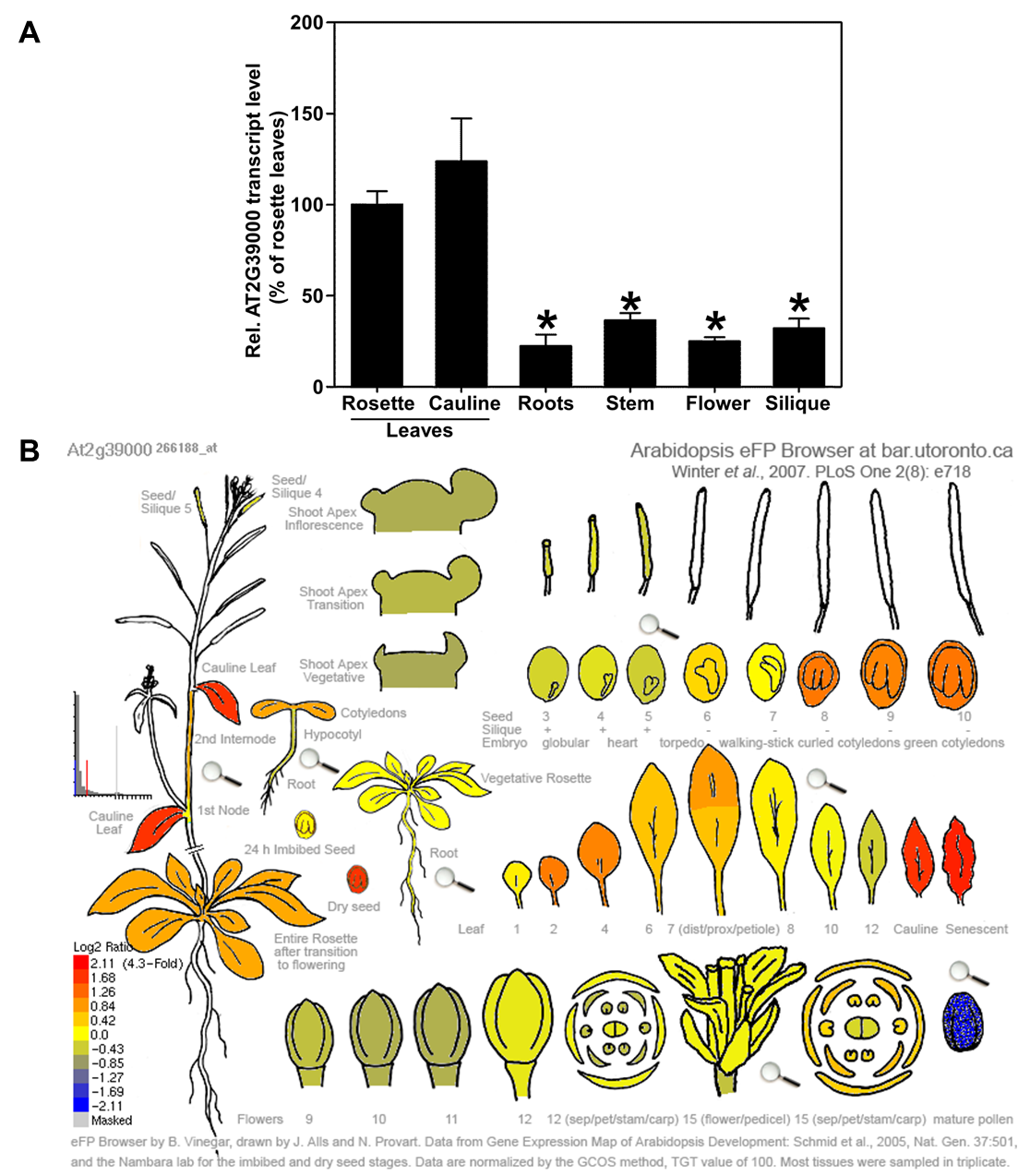


**Supplemental Figure 2. Transcription of AT2G39000**

A) Transcription of the locus AT2G39000 in various tissues of 10 week-old soil grown *Arabidopsis thaliana* eco. Col-0 plants as determined by quantitative real time PCR using the specific primers AT2G39000-F (CAAG­ATCC­CTGA­AGGA­GCGAC) and AT2G39000-R (CCGA­AACT­GTTC­AAGA­GCTT­GTG). Transcript level of rosette leaves was set to 100 %. Asterisks indicate statistically significant differences between mean of sample groups determined with the t-test (P < 0.01, N = 3). B) Relative expression of AT2G39000 according to transcriptome profiling results deposited in public available transcriptome repository databases (summarized with the eFP Browser tool (<http://bbc.botany.utoronto.ca/efp/cgi-bin/efpWeb.cgi>).

**Method description:**

Total RNA from different tissue was extracted with the PeqGOLD Total RNA Kit (Peqlab) according to the manufacturer’s protocol and transcribed into cDNA prior analysis by quantitative real time PCR as described in [[1](#_ENREF_1)]. The PP2A gene served as control for normalization: PP2A-F (5`-cttctcgctccagtaatgggatcc-3`), PP2A-F (5`-gctt­ggtc­gact­atcg­gaatgagag -3`).

**Reference:**

[1] Haas, F. H., Heeg, C., Queiroz, R., Bauer, A., et al., Mitochondrial serine acetyltransferase functions as a pacemaker of cysteine synthesis in plant cells. *Plant Physiol* 2008, *148*, 1055-1067.
