# Supplementary material for: Molecular identification and functional characterization of the first Nα-acetyltransferase in plastids by global acetylome profiling
Source: Proteomics. 2015 Jun 18;15(14):2426–35. doi: 10.1002/pmic.201500025 (PMC4692087; doi:10.1002/pmic.201500025)
Supplement: Supplementary file 3 — Figure 3 [file pmic0015-2426-sd3.docx]

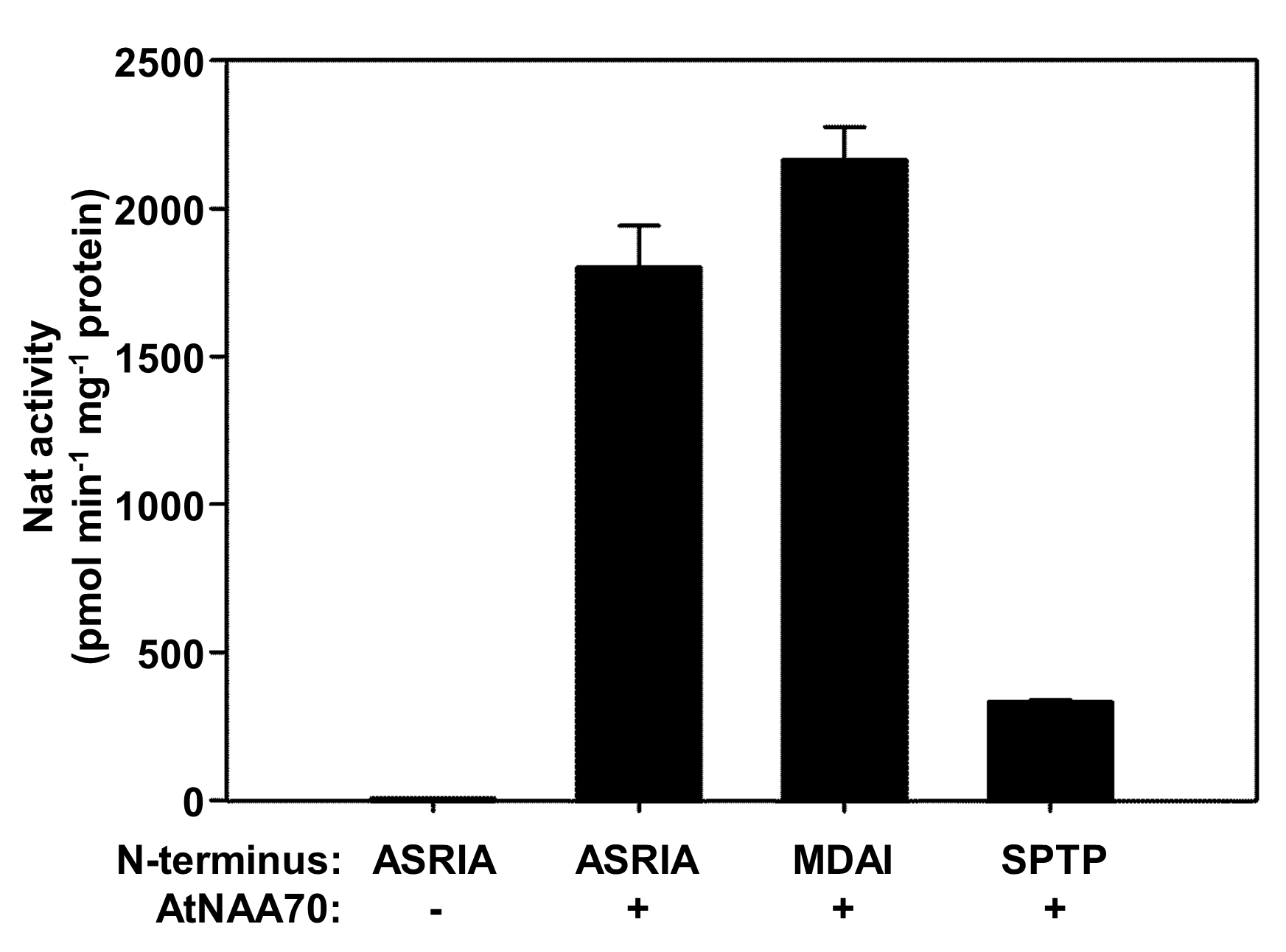


**Supplemental Figure 3. Nat activity of AtNAA70 on selected peptides**

Purified His_6_-MBP-AtNAA70 protein (5 µg, +) was incubated for 60 min in reaction buffer (50 mM Tris-HCl, pH 8.5, 10% Glycerol, 1 mM EDTA) containing 50 µM [^3^H]-acetyl Coenzyme A (7.4 GBq mmol^-1^, Hartmann Analytics) and 0.2 mM peptide (Genecust) that display different N-termini. The reaction was stopped by affinity purification of the peptides with SP-sepharose (0.15 ml) as described in [[1](#_ENREF_1)]. Incorporation of radioactive label in the peptides was determined by scintillation counting. As control for putative contamination of affinity purified peptides with [^3^H]-acetyl Coenzyme A, the ASRIA peptide was incubated for 60 min with [^3^H]-acetyl Coenzyme A without AtNAA70 (-) and affinity purified by SP-sepharose prior determination of radioactivity by scintillation counting. (N=3)

Sequence of peptides: ASRIA (N-ASRIAKDVTERWGRPVGRRRRPVRVYP-C), MDAI (N-MDAIRRRMQMLRWGRPVGRRRRPVRVYP-C) and SPTP (N-SPTPP­LFSLPR­WGRP­VGRR­RRPVRVYP-C)

**References:**

[1] Arnesen, T., Anderson, D., Baldersheim, C., Lanotte, M., et al., Identification and characterization of the human ARD1-NATH protein acetyltransferase complex. *Biochem J* 2005, *386*, 433-443.
