# Supplementary material for: Molecular identification and functional characterization of the first Nα-acetyltransferase in plastids by global acetylome profiling
Source: Proteomics. 2015 Jun 18;15(14):2426–35. doi: 10.1002/pmic.201500025 (PMC4692087; doi:10.1002/pmic.201500025)
Supplement: Supplementary file 4 — Table 1 [file pmic0015-2426-sd4.pdf]

PROTEOMICS

Supporting Information
for Proteomics
DOI 10.1002/pmic.201500025

Trinh V. Dinh, Willy V. Bienvenut, Eric Linster, Anna Feldman-Salit, Vincent A. Jung, Thierry Meinnel, Rüdiger Hell, Carmela Giglione and Markus Wirtz

Molecular identification and functional characterization of the first Nα-acetyltransferase in plastids by global acetylome profiling

© 2015 The Authors. PROTEOMICS Published by Wiley-VCH Verlag GmbH & Co. KGaA, Weinheim

Supplemental Table 1. List of primers for cloning of cDNAs

Name	Sequence	Description
At2g39000_EYFP_F	5’-GATCGGATCCATGCGGAGCACACCGTT-3’	Cloning into pFF19-EYFP
At2g39000_EYFP_R	5’-GATCGTCGACCCGAAACTGTTCAAGAGCTTG-3’	
At2g39000_pETM41_F	5’-CCATGGACGCAAGTCAAATAGTTGATCTTTTTCC-3’	Cloning into pETM41
At2g39000_pETM41_R	5’-GGATCCTTACCGAAACTGTTCAAGAGCTTG-3’	
