# Supplementary material for: Molecular identification and functional characterization of the first Nα-acetyltransferase in plastids by global acetylome profiling
Source: Proteomics. 2015 Jun 18;15(14):2426–35. doi: 10.1002/pmic.201500025 (PMC4692087; doi:10.1002/pmic.201500025)
Supplement: Supplementary file 5 — Table 2 [file pmic0015-2426-sd5.pdf]

PROTEOMICS

Supporting Information
for Proteomics
DOI 10.1002/pmic.201500025

Trinh V. Dinh, Willy V. Bienvenut, Eric Linster, Anna Feldman-Salit, Vincent A. Jung, Thierry Meinnel, Rüdiger Hell, Carmela Giglione and Markus Wirtz

Molecular identification and functional characterization of the first Nα-acetyltransferase in plastids by global acetylome profiling

© 2015 The Authors. PROTEOMICS Published by Wiley-VCH Verlag GmbH & Co. KGaA, Weinheim

Supplemental Table 2 Potential Arabidopsis Nats that are predicted to be localized in plastids

cTP, chloroplastidic transit peptide, mTP, mitochondrial transit peptide, SUBA, subcellular localization of proteins in Arabidopsis database

No.	Locus	Predicted localization according to SUBA	TargetP score	cTP cleavage site prediction by TargetP
1	At1g24040	Chloroplast	cTP : 0.963	55
2	At2g06025	Chloroplast, Mitochondrion	cTP: 0.468, mTP: 0.672	111
3	At4g28030	Chloroplast	cTP: 0.843	65
4	At2g39000	Chloroplast	cTP: 0.928	61
5	At4g19985	Chloroplast, Mitochondrion	cTP: 0.102, mTP: 0.598	93
6	At1g26220	Chloroplast, Mitochondrion	cTP: 0.695, mTP: 0.556	37
7	At1g32070	Chloroplast	cTP: 0.972	58
